# RARα1 control of mammary gland ductal morphogenesis and wnt1-tumorigenesis

**DOI:** 10.1186/bcr2724

**Published:** 2010-10-05

**Authors:** Ellen Cohn, Liliana Ossowski, Silvina Bertran, Christine Marzan, Eduardo F Farias

**Affiliations:** 1 Division of Hematology/Oncology, Tisch Cancer Institute, Mount Sinai School of Medicine, One Gustave L. Levy Place, New York, NY 10029, USA

## Abstract

**Introduction:**

Retinoic acid signaling pathways are disabled in human breast cancer suggesting a controlling role in normal mammary growth that might be lost in tumorigenesis. We tested a single receptor isotype, RARα1 (retinoic acid receptor  isotype alpha, isoform 1), for its role in mouse mammary gland morphogenesis and mouse mammary tumor virus (MMTV)-wingless-related  MMTV integration site 1 (wnt1)-induced oncogenesis.

**Methods:**

The role of RARα1 in mammary morphogenesis was tested in RARα1-knockout (KO) mice and in mammary tumorigenesis in bi-genic (*RARα1*/KO crossed with MMTV-*wnt1*) mice. We used whole mounts analysis, stem cells/progenitor quantification, mammary gland repopulation, quantitative polymerase chain reaction (Q-PCR), test of tumor-free survival, tumor fragments and cell transplantation.

**Results:**

In two genetic backgrounds (129/Bl-6 and FVB) the neo-natal *RARα1*/KO-mammary epithelial tree was two-fold larger and the pubertal tree had two-fold more branch points and five-fold more mature end buds, a phenotype that was predominantly epithelial cell autonomous. The stem/progenitor compartment of the *RARα1*/KO mammary, defined as CD24^low^/ALDH^high activity ^was increased by a median 1.7-fold, but the mammary stem cell (MaSC)-containing compartment, (CD24^low^/CD29^high^), was larger (approximately 1.5-fold) in the wild type (wt)-glands, and the mammary repopulating ability of the wt-gland epithelium was approximately two-fold greater. In MMTV-*wnt1 *transgenic glands the progenitor (CD24^low^/ALDH^high activity^) content was 2.6-fold greater than in the wt and was further increased in the *RARα1*/KO-*wnt1 *glands. The tumor-free survival of *RARα1*/KO-*wnt1 *mice was significantly (*P *= 0.0002, Kaplan Meier) longer, the *in vivo *growth of *RARα1*/KO-*wnt1 *transplanted tumor fragments was significantly (*P *= 0.01) slower and *RARα1*/KO-*wnt1 *tumors cell suspension produced tumors after much longer latency.

**Conclusions:**

In vitamin A-replete mice, RARα1 is required to maintain normal mammary morphogenesis, but paradoxically, also efficient tumorigenesis. While its loss increases the density of the mammary epithelial tree and the content of luminal mammary progenitors, it appears to reduce the size of the MaSC-containing compartment, the mammary repopulating activity, and to delay significantly the MMTV-*wnt1*-mammary tumorigenesis. Whether the delay in tumorigenesis is solely due to a reduction in wnt1 target cells or due to additional mechanisms remains to be determined. These results reveal the intricate nature of the retinoid signaling pathways in mammary development and carcinogenesis and suggest that a better understanding will be needed before retinoids can join the armament of effective anti-breast cancer therapies.

## Introduction

Retinoic acid receptors (RARs) are the main mediators of the biologic effects of vitamin A, with a long established essential role in the maintenance of the differentiated state of epithelial tissues [1]. More recently, retinoic acid (RA) and other RAR agonists were found to be growth inhibitory for cancer cell lines *in vitro *[2-7], in carcinogen-induced rodent mammary cancer models, [8-10] and in xenograft models of human cancer cell [11-13].

Because pharmacological doses of retinoids were used in the carcinogenesis studies, the question of the ability of physiological retinoid levels of vitamin A-replete animals to exert growth suppressive effects and protect epithelia from neoplastic transformation remained unanswered. It is, however, well established that the RAR-signaling pathway is defective in carcinomas of several organs, including breast, mostly due to reduced expression of *RARβ *or *CRBP-1 *[14,15]. Whether these alterations affect oncogenesis or tumor maintenance, and what might be the mechanism of these effects remains unresolved.

To address the potential role of RAR both in mammary gland morphogenesis and in modifying cancer susceptibility at physiological levels of vitamin-A, we used *RARα1 *homozygous null (*RARα1*/KO) mice and bi-genic mice generated by crossing *RARα1*/KO with MMTV-*wnt1 *transgenic mice. We found that loss of *RARα1 *produced, in pubertal glands, a highly branched ductal epithelial tree phenotype, which was epithelial cell autonomous. Because retinoids are well known for regulation of embryonic stem cells [12,16-18], and, in one case, adult HSCs [19], and because adult stem cells are known to be involved in mammary gland morphogenesis [20-27], we hypothesized that loss of *RARα *might affect the mammary stem cell compartment. Moreover, because wnt1 oncogenesis is believed to target mammary stem cells or bi-potent progenitors and might be responsible for progenitor amplification [20,28-30], we predicted that the *RARα1*/KO glands with the complex epithelial tree phenotype, will be more susceptible to wnt1-tumorigenesis.

We now show that epithelial cells derived from the highly branched ductal mammary tree of the *RARα1*/KO glands contain higher percentage of luminal progenitor cells, that they form larger primary mammospheres when cultured under adhesion-free and serum-free conditions, and that their MaSC-containing compartment is smaller than that of the wild type (wt)-glands. We further show that activation of RARα by a specific agonist inhibits primary wt-mammospheres growth. Our published work [31], showed that chronic treatment of MMTV-*wnt1 *mice with the same RARα agonist inhibited mammary tumor formation and growth. In spite of all these inhibitory effects of activated RARα we found a significant increase in tumor-free survival when mice null for *RARα1 *were crossed with MMTV-*wnt1 *transgenic mice. We propose that in vitamin-A replete conditions, *RARα *guards normal morphogenesis and influences wnt-induced tumorigenesis at least in part by maintaining a proper hierarchy of the mammary epithelial compartments.

## Materials and methods

### Animals

*RARα1-/- *mice (129/Bl-6 background) were generated in Pierre Chambon's laboratory (IGBMC, Strasbourg, France), and MMTV-*wnt1 *mice (FVB, SJL, and Bl/6 mixed background, with FVB prevalence) were generously provided by Dr. Yi Li (Baylor College of Medicine, Houston, TX, USA). The *RARα1-/+ *female mice were crossed with hemizygous male MMTV-*wnt1 *mice followed by intercrossing of F1 progeny that was *RARα1+/- *and MMTV-*wnt1 *transgenic, until sufficient mice for study were obtained. Genotyping was carried out by PCR. To obtain *RARα1 -/*- in FVB background *RARα1+/- *females were backcrossed eight times with FVB males. All animal experiments were conducted in accordance with the IACUC approved protocols following the Mount Sinai Guidelines.

### Whole mounts of mammary glands and quantification of side branching and mature terminal end buds

Mammary glands were excised, fixed in Carnoy's fixative and stained in carmine alum solution as described in reference 32 [32]. The neonatal glands were photographed and JPG files analyzed using the ImageJ software. The entire mammary tree in the abdominal number four gland/group was circumscribed and the Integrated Density of the area was measured in pixels. The branch points were counted in the three largest ducts in seven pairs (a total of 14 glands) of number four glands from the nipple to the periphery of the fat pad and the mature terminal end buds along the periphery of the fat pad.

### Mammary gland transplantation

Fragments (approximately 2 mm^3^, approximately 30,000 cells) of mammary glands of 8- to 10-week-old virgin mice (wt or *RARα1*/KO) taken from the area between the nipple and the LN, were transplanted into epithelium pre-cleared glands of three-week-old wild type or *RARα1*/KO animals, as previously described [33]. The recipient glands were excised and processed as for whole mounts eight weeks after transplantation. Seven mice were sham transplanted.

### Quantification of stem/progenitors using FACS analysis

Mammary gland numbers four and five of seven- to eight-week-old female mice were isolated, minced and digested in trypsin/collagenase. The epithelium was freed of adipocytes by centrifugation and red blood cells were lysed. The remaining cells were treated exactly as described [34]. For the characterization of MaSC-containing and luminal progenitor containing compartments, cells (1 × 10^6 ^cells/sample in duplicate) were incubated with PE or APC-conjugated anti-CD24 Ab (BD Pharmingen, San Jose, CA, USA), APC-conjugated anti-CD29 Ab (Invitrogen, Camarillo, CA, USA) and FITC-conjugated anti-CD61 Ab (eBioscience, San Diego, CA, USA). In all other flow cytometry experiments, ALDH activity was determined by the Aldefluor assay according to manufacturer's directions (Aldegen, Durham, NC, USA), followed by CD24 antibody (1:100, biotin conjugated, BD Pharmingen, secondary antibody 1:1000 streptavidin-Alexa 633 and incubation on ice for 30 minutes). A pool of cells from approximately 10 mice was routinely used for each experiment. FACS analysis was carried out using a FACScanto flow cytometer, DIVA software program for acquisition (BD Biosciences) and Flowjo (Treestar, Inc. Ashland, OR, USA) software for analysis.

### Growth of mammospheres

Mammary epithelial cells prepared as described for FACS analysis were further dissociated by pipetting and filtering through a 40 μ pore cell strainer. (Occasional small clumps of up to six cells remained). Between 2.5 to 5.0 × 10^4 ^cells were plated in ultra low adhesion 24-well plates (Corning, Corning, NY, USA) and incubated in serum-free F12/DMEM 50:50 medium (Cellgro, Mediatech, Inc., Manassas, VA, USA) supplemented with 20 ng/ml EGF and 1:50 B27 Supplement (Invitrogen, Carlsbad, CA, USA). When treatment was indicated, the agonist and antagonists were added after 8 to 10 days of incubation at 10 nM for Am580, 200 nM for at RA, and 10-fold excess of Ro41-5253 RARα antagonist, 100 nM or 2 μM, respectively, and the mammospheres were incubated for approximately eight days with partial medium and drug replenishment every two days. Mammospheres from four to eight wells were combined, allowed to settle at 1 g, dissociated with EDTA, Joklik's medium and a brief trypsin treatment, followed by Soybean trypsin inhibitor (Sigma, St. Louis, MO, USA) and cells from at least duplicate culture pools were counted either in hemacytometer or in 5 μl droplets in duplicates. For second passage the dissociated cells were re-plated as above. In some experiments mammospheres were counted before dissociation.

### Expression of RAR by Q-PCR

RNA was isolated from primary mammary epithelial cells using RNeasy kits (Qiagen, Valencia, CA, USA), according to the manufacturer's protocol. cDNA was synthesized from 2 μg of RNA using RevertAid M-MuLV reverse transcriptase (Fermetas, Geln Burnie, MA, USA). Q-PCR was performed using the 2 × SYBR Green master mix (Applied Biosystems, Carlsbad, CA, USA) with 300 nM primers and 40 ng of cDNA. To determine the fold change in expression, the C_t _value was averaged from triplicates for each sample. To normalize the *RARs *expression, triplicates for Ct from the *RAR *gene was averaged and divided by the average of the triplicate from the *GAPDH *gene. The PCR primers used were: *RARβ2 *for: 3' CTT CCT CCT GCA TGC TGC AG 5', *RARβ2 *rev: 5' GG CAC TGA CGC CAT AGT GGT A 3'; *RARγ1 *for: 3;' TGG GGC CTG GAT CTG GCT A 5', *RARγ1 *rev: 5' AT CTC CTC CGA GCT GGT GCT 3'; *RARγ2 *for: 3' CGG ACT TGA GTC TTT TGC CTG 5', *RARγ2 *rev: 5' GCT CTG TGT CTC CAC CGA TT 3'.

### Activation of wnt-pathway

Cells from three individual MMTV-*wnt1 *tumors in the first or second passage in culture, were transfected with the β-catenin reporter TOP-FLASH plasmid and renilla luciferase plasmid (pRL-SV4) in triplicates using Lipofectamine LTX (Invitrogen). After approximately 20 hr incubation, 4 μM Ro41-5253 was added and 24 hr later the cells were lysed in 1 × passive lysis buffer (Dual-Luciferase Reporter Assay System, Promega, Madison, WI, USA) and assayed for luciferase using the Dual-Luciferase reporter (DLR) Assay System (Promega).

### Identification of bi-potential cells in mammospheres

Eight-day-old mammospheres were dissociated and plated at one cell/20 μl of medium in 96-well plates (400 wells). Wells with single cells (118 total) were marked and incubated in medium with serum with weekly medium changes for a month. Of these only 21 produced colonies; these were stained with anti-CK14 (Neomarkers, Fremont, CA, USA; 1:200, secondary anti-rabbit-Alexa 488, 1:500, Molecular Probes, Invitrogen) and anti-CK18 (Sigma, St. Louis, MO, USA; 1:400, anti-mouse-Alexa 568, 1:500, Molecular Probes) and DAPI, examined under fluorescent Nikon Eclipse E600 microscope and photographed with SPOT-RTTM camera, Spot Diagnostic Instruments (Sterling Height, MI, USA) (× 200).

### Kaplan-Meier disease-free survival curves

MMTV-*wnt1 *and MMTV-*wnt1*-*RARα1*/KO mice were palpated weekly (all five-gland pairs were examined) by the same investigator. The appearance of the first palpable tumor was recorded. Mice were euthanized when tumors reached a size of approximately 1 cm^3 ^and parts of the tumors were taken for histological analysis (formalin fixation), RNA analysis (RNAlater), and in some cases for preparation of primary cultures and/or transplantation. Histological analysis of the tumors was performed by the Mutant Mouse Pathology Laboratory at UC Davis and by Mount Sinai's Center for Comparative Medicine and Surgery. Statistical analysis was done using the Log-rank test for the Kaplan-Meier survival studies and by two-way ANOVA test for the tumor growth studies. Significant differences were considered at *P *< 0.05. Specific methods are indicated in figure legends.

### Tumor transplantation

For transplantation, fragments (approximately 1 mm^3^) of MMTV-*wnt1 *and MMTV-*wnt1*-*RARα1*/KO tumors were transplanted with an implant needle (Fisher, Pittsburgh, PA, USA) into opposite sides between gland numbers three and four of anesthetized eight-week old FVB mice (four mice per experiment, repeated three times). Each transplant consisted of three randomly chosen tumor fragments from a mince. Tumor diameter was measured twice a week with a caliper. For determination of tumor latency and tumor take, primary *wnt *or *RARα1*/KO-*wnt *tumors were dissociated, the single cells counted and 10^5 ^or 10^4 ^viable cells were injected into FVB mice as above.

## Results

We studied a single *RAR *isoform knockout (KO) mice (*RARα1*/KO) because compound *RAR *mutants are embryonic or perinatal lethal whereas, with some exceptions, single isoform mutants are viable, fertile, and have a normal life span. We focused in this work on the role of the *RARα1 *isoform which, directly, or through *RARβ *expression, has been implicated in growth suppression of normal and cancerous breast cells *in vitro *[2,6,7].

### RARα controls mammary development before and during puberty

**Figure 1:**
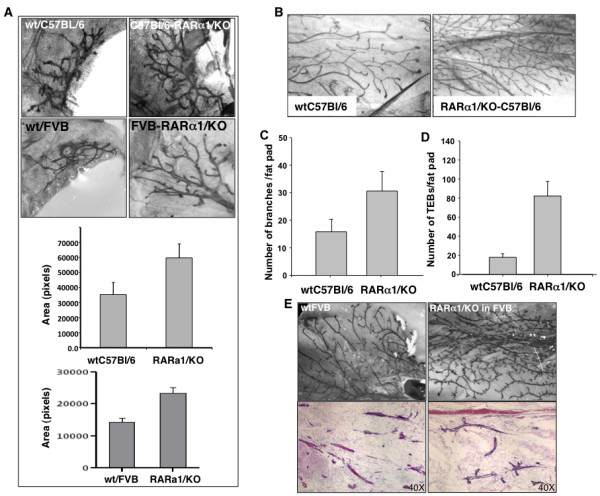
**The effect of *RARα1*/KO on neonatal and pubertal gland development**. **(a) **Effect on the size of the neonatal glands. Number 4 glands were dissected from five to six days old wt and *RARα1*/KO C57Bl/6 or FVB, four mice per group, and whole mounts were prepared as described in Materials and methods. The area taken up by the mammary tree was analyzed using the Image J software. Bars are mean and SD of four glands per group. **(b-e) **Effect on pubertal mammary tree branching morphogenesis and terminal end buds. Number 4 mammary glands from seven to eight weeks old wt or *RARα1*/KO C57Bl/6 **(b-d) **and FVB pubertal mice **(e) **were dissected and processed for whole mounts and paraffin sections. Branching points were counted along the entire length of the three longest ducts. Bars show mean and SD of seven pairs (14 total) of number 4 glands per group (*P *= 0.0001, *t*-test). The same glands were used to count the peripheral terminal end buds (TEBs) (*P *= 0.00001, *t*-test).

We analyzed *RARα1*/KO, and wild type (wt) mammary whole mounts from female mice of 129/C57Bl-6 genetic backgrounds in the first week after birth, during puberty, pregnancy, lactation, involution and post-involution and mice in FVB background after birth and during puberty. (Two genetic backgrounds were used because mammary morphology has been shown to be genotype-dependent [35,36]). A major effect of *RARα1*/KO was found in the newborn and the pubertal glands (Figure 1A-E); other developmental phases were ostensibly normal, (Results not shown). In the *RARα1*/KO neonatal glands of 129/C57Bl-6 and FVB mice the epithelial ductal tree extended approximately two-fold further into the fat pad (Figure 1A). In pubertal wt-glands, (six to eight weeks old) at diestrus the ductal tree filled most of the fat pad but several terminal end-buds (sites of active proliferation) still remained (Figure 1B, E, left panels). (The phenotype was also present in other parts of the estrus cycle, results not shown). There was a two-fold increase in branch points and a five-fold increase in the number of mature end-buds in the *RARα1*/KO fad pad (Figure 1C, D). This effect was specific to *RARα1*/KO as *RARb2*/KO mice did not show a change in branching morphogenesis (results not shown) and *RARα1*/KO produced similar branching phenotype in two different genetic backgrounds (Figure 1B, E, whole mount and histological sections). Thus, loss of a single *RAR *isoform has a major effect on the expansion of the neonatal ductal tree and branching morphogenesis in pubertal mice.

### The increased ductal branching is predominantly epithelial-cell autonomous

**Figure 2:**
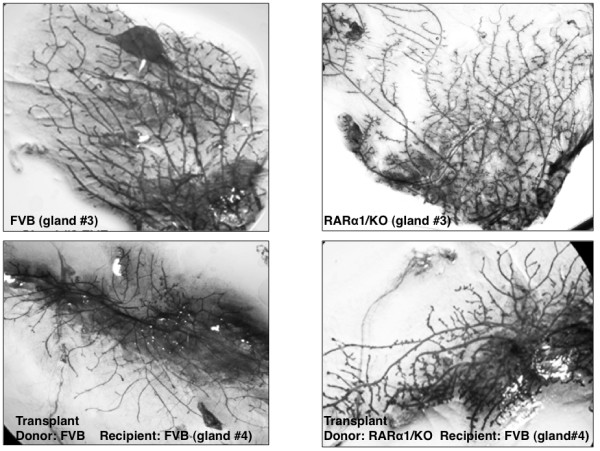
**Mammary gland transplantation**. Fragments of epithelium containing mammary glands of eight to ten weeks old virgin mice (wt or *RARα1*/KO) were transplanted into number 4 glands pre-cleared of epithelium of three weeks old wild type or *RARα1*/KO animals (see Materials and methods). At week 11 (eight weeks after transplantation) the transplant-recipient glands and intact glands number 3 from the same mouse, as control, were processed for whole mounts. >90% of wt-FVB transplants repopulated the recipient gland while <50% of the FVB-*RARα1*/KO did (see Table 1).

To exclude systemic effects of *RARα1*/KO on mammary morphogenesis, epithelium-containing mammary fragments, estimated to contain approximately 30,000 epithelial cells, were transplanted from adult FVB-wt or *RARα1*/KO donor mice into epithelium-divested, abdominal number four glands of FVB-wt and *RARα1*/KO, three-week old recipient mice such that the experiment was always internally controlled. The transplanted number four glands and the number three glands from the recipient mice, used as internal controls, were excised eight weeks later, and their phenotype examined in whole mounts. We found (Figure 2) that donor tissue from wt and *RARα1*/KO mice was able to repopulate the recipient glands; the highly branched ductal tree of the *RARα1/*KO donor was fully reconstituted in a wt-FVB recipient, indicating that the phenotype was epithelial cell autonomous. The "penetrance" of the KO-phenotype was slightly greater when both the donor and the recipient mouse were of the KO-type, (75% KO-phenotype in wt-recipients and 86% in *RARα1*/KO recipients, Table 1), suggesting possibly a minor systemic effect. The majority (86% and 87%) of wt-FVB transplants had the expected wt-phenotype in the FVB-wt and *RARα1*/KO recipients, (Table 1).

**Table 1:** Epithelial-autonomous phenotype of *RARα1*-null phenotype

Categories	*RARα1*-null to FVB	*RARα*-null to *RARα*-null	FVB to FVB	FVB to *RARα*-null
Total number transplanted	17	18	30	17
Successful gland repopulation (%)	47.0	39.0	93.3	94.1
Expected phenotype (%)	75.0^1^	86.0^1^	86.0^2^	87.0^2^

^1 ^Increased ductal tree branching, more total and mature TEBs, ^2 ^Normal branching phenotype.

### Primary RARα1/KO mammary gland mammospheres contain more cells

**Figure 3:**
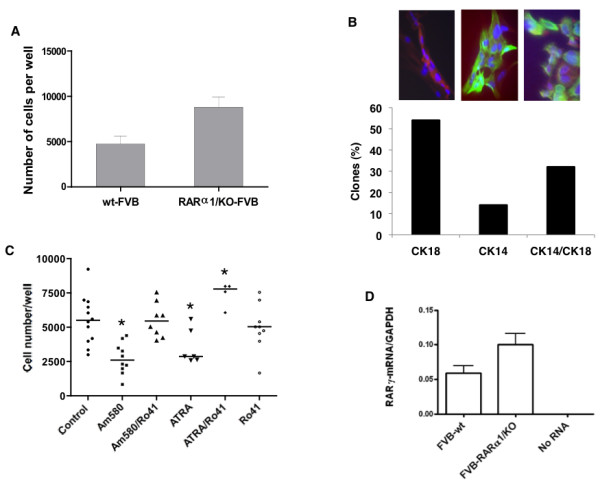
**Growth, composition and regulation of primary mammospheres**. **(a) **Comparison of mammosphere growth. Primary cells obtained from wt and *RARα1*/KO mammary glands, were inoculated at 2.5 to 5 × 10^4 ^under conditions of ultra-low adhesion and serum-free medium and eight days later, the mammospheres were disassociated and the cells counted (see Materials and methods). The bars show mean (4,750 and 8,833 cells respectively, and SD of three individual experiments and three to four samples per group; *P *= 0.04 by unpaired *t*-test. **(b) **Bi-potential cells in mammospheres. Primary mammary epithelial cells were incubated for eight days until most single cells died; mammospheres were dissociated, inoculated into 96 wells, at 1cell/well (see Materials and methods) and when colonies formed stained for CK18 and CK14. (Red - CK18; green - CK14; blue - DAPI). Total number of colonies stained = 21. Scale bar = 10 um. **(c**) Regulation of mammosphere growth by retinoids. Cells isolated from mammary glands of seven- to eight-week old FVB mice were inoculated under conditions of mammosphere formation and eight to ten days later were treated with 10 nM of Am580, 200 nM of atRA, and/or 10-fold excess (100 nm and 2 μM, respectively) of RARα antagonist, Ro41-5253 for seven to eleven days. Each symbol represents an individual well; there were two to four wells per experiment, and a total of four experiments. Kruskal-Wallis test *P *= 0.0002; *indicates significance at *P *< 0.05 by Dunn's Multiple Comparison Test. **(d) ***RARγ1 *expression. RNA was extracted from freshly isolated and partially purified mammary epithelial cells and wt and *RARα1*/KO, and subjected to Q-PCR analysis as described in Materials and methods. The bars show mean of three experiments, two samples in each.

We wondered whether the increased branching and, presumably, the increased epithelial content of the *RARα1*/KO gland is a result of a larger stem/progenitor cell pool. As a first test of this idea we prepared single cell suspensions of partially purified mammary epithelial cells from glands of seven- to eight-week-old wt or *RARα1*/KO mice, seeded them under mammospheres conditions, dissociated the mammospheres after eight days of attachment-free growth and counted the individual cells [37]. As shown in Figure 3A, in four independent experiments, wells seeded with *RARα1*/KO derived epithelium produced mammospheres that, upon dissociation, had approximately two-fold more cells (*P *= 0.01) than those obtained from wt-epithelium. The difference in the number of mammospheres, which were difficult to count, was slightly and not significantly lower in the *RARα1*/KO cultures. We were unable to efficiently passage any mammospheres beyond Passage 3.

Although, mammospheres are considered to be useful culture correlates to stem cells, it appears that only a small percent of mammosphere cells have repopulating *in vivo *potential, and their derivation and composition, especially in the mouse mammary, is still debated [25,38]. We characterized the primary mammospheres for the presence of bi-potential progenitors [39]. This was done by inoculating single cells obtained from primary mammospheres into small wells, and when colonies were established, staining them for cytokeratin CK14 (myoepithelial cells) and CK18 (luminal cells). Of a total of 118 wells with single cells which started dividing, only 21 (18.6%) formed, after a month of incubation, large enough colonies to be stained with antibodies to CK14 and CK18. We found (Figure 3B) that only 30% of the colonies produced by single cells were bi-potential, with individual cells within the colony expressing either CK18 or CK14; 55% of the colonies expressed exclusively CK18, (luminal), and 15% exclusively CK14 (myoepithelial) markers. Thus more than 80% of cells in mammospheres are unable to produce colonies in adherent culture, and of those cells that make colonies, only approximately 30% are bi-potential.

### RAR-activation is involved in growth of primary mammospheres

To better link the increase in cell number in the *RARα1*/KO-derived mammospheres with the RAR pathway, wt-mammary epithelial cells were allowed to form mammospheres and were either left untreated or were treated for eight days with 10 nM Am580, (a RARα agonist) or with Am580 combined with a 10-fold excess of Ro41-5253, a RARαantagonist, at which time the mammospheres were enzymatically dissociated and single cells counted. We found that activation of RARα by Am580 reduced mammosphere growth by 50%, while RARα-antagonist in presence of Am580, completely reversed this inhibition (Figure 3C), mimicking to some degree the loss of *RARα1*. We also found that mammospheres treated with 100 nM atRA, an agonist for all three RARs, in presence of the specific RARα antagonist, boosted the cell number in mammospheres even above that of untreated controls (Figure 3C). Comparison of *RAR *expression by Q-PCR in RNAs extracted from wt and *RARα1*/KO epithelial cells isolated directly from the mammary glands, showed that all three *RAR *isotypes were present, but while there was no difference in *RARα1/2 *and *RARβ2 *expression (results not shown), in three independent tests, the *RARγ1 *expression was increased by almost two-fold in the *RARα1*/KO cells (Figure 3D). Our previous work [31], showing a pro-proliferative role of RARγ suggests that, in addition to blockade of the RARα, the increased RARγ1 might have contributed to the cell increase in mammospheres treated with atRA and Ro41-5253 (Figure 3C). Whether this effect was mediated through a specific effect on the progenitors remains to be determined.

### RARα1 loss affects mammary epithelial hierarchy and mammary repopulating efficiency

**Figure 4:**
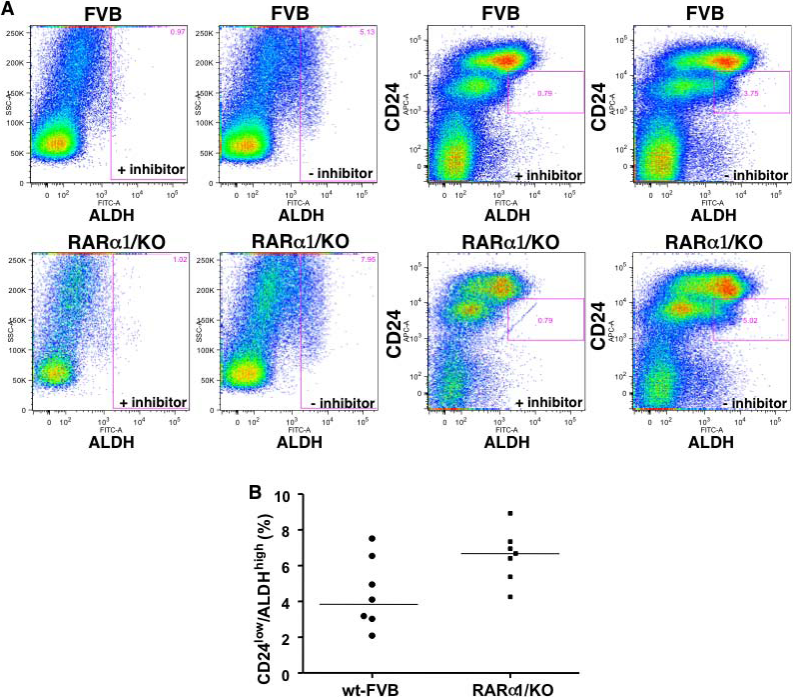
**Characterization of the stem/progenitor cell compartment of wt-FVB and *RARα1*/KO**. **(a) **Representative histograms of flow cytometry analysis of CD24^low/^ALDH^high ^compartment in mammary epithelium. Primary mammary epithelial cells (approximately1 × 10^6^) were incubated with ALDH substrate with inhibitor (first and third from left upper and lower panels) or without inhibitor (second and fourth from left upper and lower panels) followed by incubation with anti-CD24 Ab and detected with secondary Ab (APC-conjugated) as described in Materials and methods. Inclusion of the DEAB inhibitor reduced the ALDH activity by 82 and 87%, in FVB and *RARα1*/KO cells, respectively. Gated: CD24^low^/ALDH^high ^**(b) **Summary of seven individual experiments. Experiments were performed as in A, shown are median, 3.9% and 6.7% respectively, (mean 4.4% and 6.6%, respectively, *P *= 0.04, unpaired *t*-test).

We first tested the effect of *RARα1*/KO on the stem/progenitor compartment of the mammary epithelium. This was done by determining the percent of cells expressing low levels of CD24 antigen (CD24^low^) and showing high ALDH activity (ALDH^high^) in cell suspensions of partially purified mammary epithelial cells from glands of seven- to eight-week-old wt or *RARα1*/KO mice. These markers were shown to define a population encompassing the mammary repopulating cells but also progenitors [24,34,40]. A representative FACS analysis (Figure 4A) shows that the *RARα1*/KO epithelium contains more CD24^low ^ALDH^high ^cells than wt-epithelium. In a total of seven experiments (Figure 4B), we found a statistically significant, (*P *= 0.01), median 1.7-fold increase in these cells in *RARα1*/KO glands. To test which specific subpopulations were affected by the loss *of RARα1*, we used an additional surface marker, (CD29). Populations of CD24^low ^cells with high expression level of CD29 (and/or CD49f) represent the MaSC-containing compartment, while CD24^high^/CD29^low ^are considered to be luminal progenitors [25]. As shown in Figure 4C, the *RARα1*/KO mammary epithelium contained greater percentage of progenitors (CD24^high^/CD29^low) ^but fewer MaSCs (CD24^low^/CD29^high^) than wt glands. Importantly, the ratio of progenitors/MasSCs in the *RARα1*/KO epithelium was 1.9, while in the wt glands it was only 0.8. Both progenitors and MaSC-compartment were positive for CD61 (results not shown).

**Figure 5:**
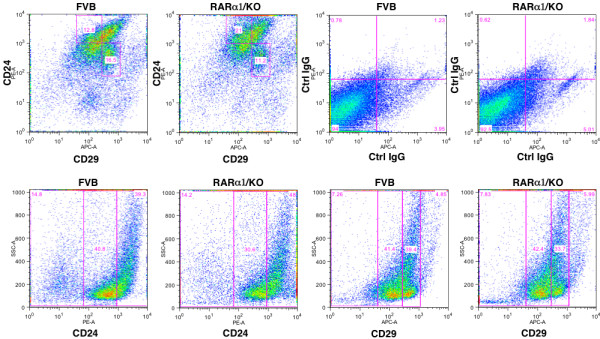
**Characterization of MaSC-containing and luminal progenitor containing compartments**. Mammary epithelial cells isolated as in Figure 4A, were incubated with PE-conjugated anti CD24 Ab, or APC-conjugated anti CD29 Ab and analyzed as described in Methods. Gated: upper left (FVB) and upper second from left (*RARα1*/KO) for CD24^high^/CD29^low ^(luminal progenitors, upper left square) and for CD24^low^/CD29^high ^(MaSC, lower right square). The third and fourth upper squares-negative controls, cells incubated with conjugated IgGs. The results shown are of one experiment, which was repeated once.

The increase in the MaSC-containing compartment of wt glands suggest that these epithelium might be more effective in repopulating epithelium-divested mammary glands. To test this we transplanted mammary fragments containing approximately 30,000 cells which, according to published reports [25] should contain 1 to 10 stem cells). As shown in Table 1, while >90% of the wt-donors produced successful transplants, only 47% of *RARα1*/KO mammary fragments were successful when transplanted into wt-mice. (The rate went down to 39% when the recipients were *RARα1*/KO mice). Thus, similar size fragments of *RARα1*/KO glands contain proportionally fewer stem cells, or contain stem cells with impaired activity. Although, preliminary, this finding is in agreement with the increased MaSC-compartment of the wt-mammary (Figure 5) and suggests that loss of *RARα1*/KO may alter the normal hierarchy of mammary epithelium.

### Loss of RARα1 delays mammary tumor development in MMTV-*wnt1 *mice, slows the growth and increase latency of transplanted tumors

**Figure 6:**
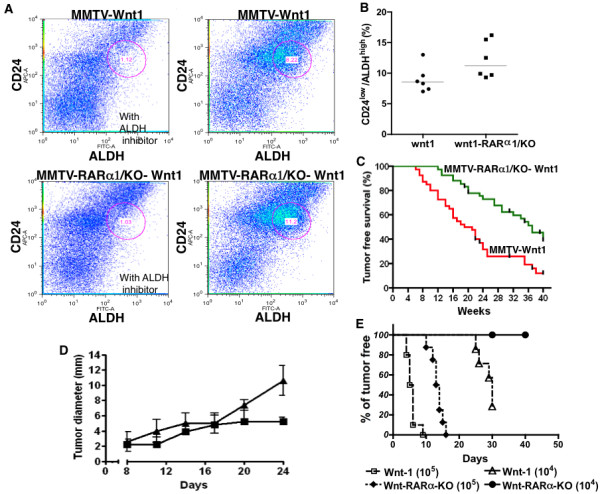
**Wnt1-tumorigenesis and wt and *RARα1*/KO transplanted tumor growth**. **(a**) Representative histograms of flow cytometry analysis of CD24^low/^ALDH^high ^cells in FVB-*wnt1 *and FVB-*wnt1*-*RARα1*/KO- mammary glands. Primary mammary cells were isolated from glands of seven-week-old mice and processed for flow cytometry analysis as described in Figure 3. **(b) **Summary of flow cytometry experiments. The experiments were carried out as in A; each dot represents individually processed sample. Statistical analysis, two way ANOVA, *P *= 0.0025. **(c) **Tumor-free survival (Kaplan-Meier curve). Forty female mice transgenic for Wnt1 and wt-RAR and 42 *RARα*/KO were allowed to age and were examined at regular intervals for the appearance of a palpable tumor nodule. The difference in disease-free survival (percent of tumor-free mice), plotted as a function of post-natal age (Kaplan-Meier curve) was statistically significant (*P *= 0.0002). **(d) **Transplanted MMTV-*RARα1*/KO-wnt1 tumor fragments have slower growth rate than MMTV-*wnt1 *tumors. Fragments of randomly chosen pairs of MMTV-*wnt1*/wt and MMTV-*wnt1*/*RARα1*/KO tumors were transplanted into the opposite flanks of wt-FVB hosts. The tumors were measured every three days. The bars show mean and SE (*n *= 12 per group, *P *= 0.01. two-way ANOVA test). **(e) **Inoculation of MMTV-*wnt1*/*RARα1*/KO cell suspensions produces tumors after longer latency. Cell suspensions (10^5 ^and 10^4^) prepared from a pair of similar size MMTV-*wnt1*/*RARα1*/KO and MMTV-*wnt1 *tumors were inoculated into eight to ten FVB-mice, (as in D) and appearance of palpable tumors was recorded and plotted as fraction of tumor-free mice vs. days post-inoculation (Kaplan-Meier).

The target cell of wnt1-oncogenesis in mammary gland has been identified as the stem cell, the progenitor or both. It has been also shown that wnt1 can expand and change the stem cell compartment [20,28,41,42]. We confirmed, using CD24^low^/ALDH^high ^as markers, that epithelial cells from MMTV-*wnt1*-glands had a much higher proportion of these cells (mean 8.2% Figure 6A) than wt-glands and their content was further increased (mean 11.2%, Figure 6A and 6B) in the bi-genic (*RARα1*/KO × MMTV-*wnt1*) mammary glands. We noted that unlike the wt-cells, which had distinct high and low CD24 populations, the wnt1 expressing cells had a more homogenous and lower expression of CD24, precluding their further sub-division based on this marker. This change has been seen previously [20,28,41,42]. Since both stem cells and progenitors are considered to be wnt1-targets, the changes we observed in these compartments made it difficult to predict what effect the loss of *RARα1 *will have on mammary tumorigenesis. To test this, mammary glands of 40-MMTV-*wnt1 *and 42-MMTV-*wnt1*-*RARα1*/KO young female mice were palpated weekly for 40 weeks, and the appearance of palpable tumors was recorded. Mice were sacrificed and the tumors collected when they reached the size of approximately 1 cm^3^. Kaplan-Meier analysis showed that the overall tumor-free survival was significantly (*P *= 0.0002) longer in the bi-genic mice and that, compared to MMTV-*wnt1 *mice, approximately three times as many MMTV-*wnt1*-*RARα1*/KO mice were tumor-free at the end of the 40^th ^week follow up (Figure 6C). H&E stained sections of the wnt1 and bi-genic tumors had similarly typical wnt-histology (results not shown). This result suggests that the reduction in the MaSCs containing sub-population, by reducing the target population, might be one mechanism through which *RARα1 *loss slows down wnt1-oncogenesis.

Because these experiments were done in mice with germ line KO of RARα1, to link the effect to the tumor itself, we transplanted fragments of *wnt1*-*RARα1*/KO and wnt-tumor on the contralateral sides of four wt-mice between the third and the fourth mammary gland. The mice were palpated weekly and once tumors were detected, their diameters were measured every three days. Figure 6D shows that between Day 8 and 17 the wt-wnt1-derived tumors showed a small growth advantage over the *wnt1*-*RARα1*/KO tumors, but in the next seven days they grew exponentially, reaching the weight of 500 mg, (calculated from the measurements of tumor diameter), while the growth of *wnt1*-*RARα1*/KO tumor leveled off at about 60 mg. Thus, even in a *RAR*-intact systemic environment, the growth of transplanted tumor cells lacking *RARα1 *was delayed.

To measure tumor latency, 10^5 ^and 10^4 ^single cell suspensions obtained from wnt1 and *wnt1-RARα1*/KO tumors were injected as above and the time of palpable tumor appearance was recorded. Figure 6E (Kaplan Meier plot) shows that, at both cell concentrations, cells derived from the *wnt1*-*RARα1*/KO tumors had much longer latency.

## Discussion

Our results provide evidence that at physiological levels of vitamin A, a single *RAR *isoform, *RARα1 *participates in the control of normal branching morphogenesis of the pubertal mammary epithelial tree. This is evidenced by the excessive side budding and secondary branching observed in mice of two genetic backgrounds when *RARα1 *is knocked out (Figure 1). Although, control of mammary morphogenesis is complex and can be driven by both systemic [43-45] and local [46-48] effects, our mammary transplantation experiments results (Figure 2 and Table 1) indicate that in this capacity, *RARα1 *functions in a predominantly epithelial-cell autonomous fashion. In addition to the effects in the pubertal gland, we found that loss of *RARα1 *causes doubling of the rudimentary mammary tree in the neonatal gland (Figure1), suggesting a possible role for this receptor in embryonic mammary development. A similar branching phenotype has been described in transgenic mice overexpressing a DN-*RAR *mutant, which blocks all retinoid signaling [49], but, to the best of our knowledge, ours is the first described link between a single *RAR *isoform and mammary epithelial growth and morphogenesis in vitamin A replete animals.

Based on the increased epithelial cellularity of the KO-glands and published data implicating *RARα *in anti-cancer activity, we expected that the *RARα1*/KO mice crossed with the MMTV-*wnt1 *mice will be more susceptible to wnt1-induced tumorigenesis. Moreover, activation of retinoid signaling has been shown to inhibit the wnt1 pathway in cell culture [50,51]. Indeed, treatment of cells derived from three individual wnt1-tumors and transfected with a reporter for wnt1 pathway activation (TCF/β-catenin activity), when treated with an RARα antagonist, Ro415253 produced a significant (*P *= 0.01) two-fold increase in the reporter (luciferase) activity (results not shown). However, unexpectedly, we found that MMTV-*wnt1*-*RARα1*/KO mice had significantly longer tumor-free survival than MMTV-*wnt1 *wt-mice (Kaplan Meier analysis, Figure 6). Moreover, in three independent experiments fragments of *wnt1*-*RARα1*/KO tumors grew much more slowly than fragments of wnt1 tumors when implanted into contra lateral sides of the same mouse, and single cells suspensions obtained from *wnt1*-*RARα1*/KO tumors showed much longer latency.

How is it possible that active RARα1 which prevents apparent epithelial hyperplasia and blocks wnt pathway activity, also allows a more efficient wnt1-induced oncogenesis? The oncogenic targets of wnt1 are believed to be progenitor/stem cells [28,29] and wnt signaling might have a role in mammary stem cells self-renewal [24]. The uncertainty regarding the precise target comes from the difficulty in comparing stem cell compartments in normal mammary and in wnt1 induced tumors. The situation is further complicated by the findings [42] that in the pre-neoplastic stage constitutive wnt1 signaling perturbs the epithelial hierarchy, leading to the emergence of aberrant multipotential stem-like cells in the committed luminal cell fractions. How can then our result fit into this complex scheme? We showed that pre-malignant *wnt1-RARα1*/KO-glands have the highest content of ALDH^high^/CD24^low ^cells and that pubertal *RARα1*/KO mammary gland (without wnt1-expression) contains 1.7 times more of these cells than wt-mammary (Figures 4 and 5). Cells isolated from *RARα1*/KO and mammospheres produced by the *RARα1*/KO cells form larger mammospheres (Figure 3), possibly because the progenitors might proliferate more rapidly. These progenitors were shown to be elevated during puberty, concomitant with an increased ductal branching and elongation, a phenotype that is enhanced in the *RARα1*/KO mice [52]. At the same time, however, we found a reduction in MaSc-enriched compartment and a reduction in repopulation efficiency of *RARα1*/KO mammaryfragments (Figure 2 and Table 1). Although, we did not perform consecutive *in vivo *passages of the transplants, the diminished capacity of the *RARα1*/KO fragments to repopulate, suggests that they contain fewer stem cells or, at the least, they contain stem cells with diminished activity.

That RARα1 keeps in check the proliferative capacity of the progenitors in the pubertal gland and, that its loss leads to the enlargement of the progenitor compartment, fits with the established anti-proliferative role of RAR and retinoids. For example, p27^kip^, a protein that accumulates in response to RAR activation, can limit the self-renewal of some adult tissue progenitors [53]. It is also possible, that in addition to removing a block to proliferation, loss of *RARα1*/KO provides an indirect proliferative stimulus mediated through RARγ1. We showed that *RARα1*/KO epithelium has higher levels of *RARγ1*-mRNA (Figure 3D), and that this isoform has pro-proliferative activity in mammary cells [31]. Moreover, wt-mammospheres treated simultaneously with atRA and an RARα antagonist, a combination that allows RARγ (and β) activation (Figure 3C), yielded the highest numbers of cells.

Overall, our current data suggest that the profound reduction in mammary repopulating activity combined with the predominance of progenitors over MaSC-containing compartment in the *RARα1*/KO, by reducing wnt1 target might contribute to the delay in wnt1-tumor development. The mechanism for this is unknown, but it has been shown that PTEN loss in HSCs causes a transient expansion of the stem cell pool followed by its depletion [54,55]. We have shown that RAR activation inhibits PI3K activity, thus loss of RARα1 might possibly cause a similar effect to that of PTEN deletion. In an elegant study Purton *et al*. [19] have shown that loss of *RARα1*, but not RARα, results in reduced numbers of HSCs and increased numbers of more differentiated progenitor cells in the mutant mice. Although, in the mammary it is the RARα that appears to be involved, different RAR isotypes are known to have tissue-specific roles [17,56].

Because wnt1 pathway activation is involved in human breast cancer [57], and RARα has a proven role in breast cancer, the mechanism connecting RARα1 to control of wnt-tumorigenicity is worthy of further study. It remains to be determined whether a reduction in stem cell level in *RARα1*/KO mice translates into reduced cancer stem cell level, what is the mechanism responsible for this reduction and whether it is the cause of increased tumor-free survival and a delay in growth of transplantable tumors.

In summary, we showed that loss of *RARα1 *leads to reduced mammary stem cell content and an increase in wnt1-tumor free survival in mice. Under physiological conditions RARα1 signaling appears to control the content of mammary stem/progenitor cell compartments and affect the proper morphogenesis of neonatal and pubertal mammary gland.

## Conclusions

The established anti-proliferative effect of retinoids on breast cancer in culture and in selective *in vivo *models did not translate, so far, into a successful anti-breast cancer therapy. Our results led us to conclude that retinoic acid receptor isotypes might have unique functions in normal development and in oncogenesis and that some of the functions can only be discerned *in vivo *under physiological vitamin-A replete conditions. For example, RARα1 appears to control the normal neonatal and pubertal morphogenesis of the mammary gland, most likely by controlling the size of the mammary stem cells (MaSCs) and progenitor compartments. Importantly, efficient wnt1-induced oncogenesis appears to be dependent on properly maintained MaCS/progenitor hierarchy. The RARγ isotype most likely has an opposing function. These unexpected results, together with recent publications by us and others, reveal the complexities of the RAR-signaling networks and suggest that to be successful, anti-breast cancer therapy will have to consider these newly identified intricacies.

## Abbreviations

ALDH: aldhyde dehydrogenase; CD24low: heat stable antigen low; CK14: cytokeratin 14; CK18, cytokeratin 18; CRBP-1: cellular retinol binding protein 1; FACS: fluorescent activated cell sorting; H&E: hematoxilin and eosin; HSC: human stem cells; LN: lymph node; MaSC: mammary stem cells; MMTV: mouse mammary tumor virus; Q-PCR: quantitative polymerase chain reaction; RA: retinoic acid; RAR: retinoic acid receptor; RARα1: retinoic acid receptor isotype alpha isoform 1; Wnt1: wingless-related MMTV integration site 1.

## Competing interests

The authors declare that they have no competing interests.

## Authors' contributions

EC assisted in collection and assembly of data, data analysis and editing of the manuscript. LO assisted in conception and design, collection and assembly of data, data analysis, and manuscript writing. SB assisted in collection and assembly of *in vivo *data. CM assisted in collection, assembly and analysis of morphogenesis data. EFF assisted in conception and design, collection and assembly of data, and data analysis. All authors read and approved the final manuscript.
